# Exploring Simplified Methods for Insect Chitin Extraction and Application as a Potential Alternative Bioethanol Resource

**DOI:** 10.3390/insects11110788

**Published:** 2020-11-12

**Authors:** Mahmoud Kamal, Eslam Adly, Sulaiman Ali Alharbi, Amany Soliman Khaled, Magda Hassan Rady, Nevin Ahmed Ibrahim

**Affiliations:** 1 Department of Entomology, Faculty of Science, Ain Shams University, Cairo 11566, Egypt; mkasu@sci.asu.edu.eg (M.K.); amany1962@hotmail.com (A.S.K.); drmagdaradi@yahoo.com (M.H.R.); 2 Department of Botany & Microbiology, College of Science, King Saud University, P.O Box 2455 Riyadh 11451, Saudi Arabia; sharbi@ksu.edu.sa; 3 Department of Microbiology, Faculty of Science, Ain Shams University, Cairo 11566, Egypt; nevinibrahim@sci.asu.edu.eg

**Keywords:** chitin extraction, bioethanol, biomass conversion, biofuel, submerged fermentation, American cockroach, *Mucor circinelloides*

## Abstract

**Simple Summary:**

The studies on chitin utilization as a source for bioethanol production are still very few. The present study explores some simple methods for insect chitin extraction and application in bioethanol production. Using insect chitin in bioethanol production, may help decreasing the dependence on energy crops as a carbon source for bioethanol. Fungal strains of *Mucor circinelloides* were reported previously to bio-convert chitin directly to ethanol in submerged fermentation systems. In our study, we explored the bioconversion of insect chitin to bioethanol using two different strains of *Mucor circinelloides* in submerged fermentation systems. An insect-isolated *M. circinelloides* strain was found to bio-convert the extracted chitin directly to ethanol in submerged fermentation system. The source of strain isolation and the pH of the production medium were showed to influence the chitin bioconversion directly to bioethanol. All fermentation processes can be conducted easily, using the whole growing microorganism instead of using purified enzymes. These results highlight the insect biomass as a potential new, cheap and renewable source for bioethanol production simply, using a potent insect-isolated *M. circinelloides* strain.

**Abstract:**

Chitin, the second most plentiful biopolymer in nature, is a major component of insect cuticle. In searching for alternative resources for fossil fuels, some fungal strains of *Mucor circinelloides* from an insect-source were found to produce bioethanol directly using insect chitin as a substrate. Herein, simplified methods for insect chitin extraction and application as a substrate in submerged fermentation for bioethanol production were explored. Chitin of the American cockroach (*Periplaneta americana* (L.)) was isolated by refluxing the cockroaches dried exoskeletons with 4% NaOH. The purity of the extracted chitin was assessed to be high when the physicochemical properties of the extracted chitin matched these of commercially available crab and shrimp samples. The extracted chitin was employed as a substrate in submerged fermentation using two strains of *M. circinelloides*. One of these, strains *M. circinelloides* 6017 showed immense potential for bioethanol production directly. It could to bio-transform 15 g/L of colloidal chitin directly to 11.22 ± 0.312 g/L of bioethanol (74% of the initial chitin mass) after 6 days of incubation. These results confirm the possibility of using insect biomass as a potential alternative resource for bioethanol production in a simple manner thus contributing to the creation of an alternate energy source.

## 1. Introduction

Climatic problems caused by carbon emissions from burning fossil fuels and depletion of these energy resources are one of the most threats that urge humanity to search for clean, renewable and low-cost alternative energy resources [1,2]. Among many renewable energy resources, biofuels have been introduced to reduce the dependence on fossil fuels; thereby, reducing atmospheric carbon emissions [3]. Biogases, biodiesel and bioethanol are the main forms of biofuels that can be derived from natural renewable biomass such as plants, algae and organic wastes [4,5]. Bioethanol, which is produced mainly from the conversion of renewable cellulose biomass has been used widely in the USA and Brazil as a new clean energy resource [5,6]. Energy crops, including sugarcane, sweet sorghum, soybeans and corn are the most widely used resources for bioethanol and biodiesel production. However, these conventional energy crops are unable to meet the global demand for bioethanol production because of the in cost, time and vast land required for their production as well as their primary value as human feed.. Therefore, searching for a new alternative bioethanol resource has become a global priority [6,7,8].

The Structural aminopolysaccharide, chitin is made from monomeric unit N-acetyl-d-glucosamine (NAG) [9]. Chitin and NAG were found to be readily available for bioconversion and production of bioethanol and hydrogen gas using different microorganisms [8,10,11,12,13]. Mucor, a dimorphic Zygomycota, was found to produce bioethanol from various carbon sources like hydrolysates of lignocellulose [14,15]. Besides, some Mucor species were proved to produce chitin-degrading enzymes [16,17]. Following these reports pointed out strains of *Mucor circinelloides* able to produce bioethanol directly using chitin as a substrate in the submerged fermentation systems [8]. Chitin is the most abundant renewable biopolymer in nature after cellulose and has been found in a broad diversity of unicellular [18] and multicellular organisms [19,20,21]. The renewal of the chitin biomass is estimated in the order of 10^10^ to 10^11^ tons annually since it is the major structural polysaccharide in the cell wall of most fungi, yeasts, shells of crustaceans and insects’ exoskeleton [22,23]. Hence, chitin and its monomeric unit were proved to be available for bioconversion to bioethanol [8,13] thus, applying chitin in bioethanol production could decrease the dependence on energy crops as a source and saving it for human feed.

Crab and shrimp exoskeletons are considered the main source for commercially produced chitin [22]. Although, chitin is the main structural polysaccharide of insect exoskeleton only limited numbers of insect species have been documented as sources for chitin extraction [24]. Insect species diversity represents more than 90% of the animal kingdom, they are an enormous untapped and available biomass [24,25]. In nature, many species of scavenger insects contribute to the recycling of organic matter, thus, they can be used to degrade organic waste and to obtain economically viable biomass. Additionally, large-scale insect breeding can occur indoors such as warehouses. Thus, there is no need to use large land areas as in the production of energy crops or to use large water areas for the production of biofuels from microalgae and crustaceans [26].The American cockroach, *Periplaneta americana* (L.) is one of the most familiar worldwide omnivorous insects [27]. It is a scavenger and consumes decaying organic matter. It can attain enormous numbers with rapid reproduction and development in small, moist and warm areas [28]. So, *P. americana* can be a promising potential alternative source for chitin production [28,29], consequently, for the production of biofuels. In our preliminary study, we tried to simplify the traditional methods of insect chitin extraction and using it as a potential alternative source for bioethanol production through fungal bioconversion. Chitin was extracted from the dried exoskeleton of the American cockroach, *Periplaneta americana* in one step and used in a submerged fermentation system for bioethanol production. Two strains of *M. circinelloides* of different isolation sources were tested to assess the influence of isolation source on the direct chitin bioconversion process. Which draws a new strategy and possibility of using insect chitin in bioethanol production with reduced cost and wastes of chitin extraction and fermentation processes.

## 2. Materials and Methods 

### 2.1. Cockroach Chitin Extraction and Characterization

#### 2.1.1. Chitin Extraction

The American cockroach, *P. americana* samples were obtained from various localities in Cairo, Egypt by hand collecting. A mixture of adults from these collections was used in all experiments. The cockroaches were killed using chloroform-wetted cotton in a tightly sealed container. The whole cockroach bodies were then air-dried for one week. Experimentally, 200 g of these air-dried exoskeletons were refluxed with 2 L of 4% NaOH for 48 h at 90 °C until a brownish colored alkaline-based extract was obtained. The extracted chitin was filtered and rinsed using tap water until the pH value reached 7 and the brown color of the extract completely disappeared. Finally, the colorless one-step extracted *P. americana* chitin (OSPC) was dried at 150 °C for 2 h and ground for further characterization.

#### 2.1.2. Characterization of the Extracted Cockroach Chitin

The physicochemical properties of the OSPC were characterized by Fourier Transform Infra-Red spectroscopy (FTIR), X-ray diffraction (XRD) and elemental analysis. Two commercial samples, shrimp chitin (Qualikems fine chemicals Pvt. Ltd., New Delhi, India) and crab chitin (Win lab Laboratory Chemicals, Leicester Shire, United Kingdom), were also characterized using the same techniques as references for comparison with the OSPC.

##### Fourier Transform Infrared Spectroscopy (FT-IR)

The infra-red spectra of the three chitin samples were recorded from 4000 to 400 cm^−1^ in KBr pellets by infrared spectrophotometry (WGH-30/30A, ©Angstrom Advanced Inc., Stoughton, MA, USA) at the Central Laboratory, Faculty of Science, Ain Shams University, Cairo, Egypt.

##### X-ray Diffraction (XRD)

The X-ray diffraction patterns of the three chitin samples were determined by a D/Max-RA diffractometer (Rigaku, Tokyo, Japan) with Cu radiation, at The Central Metallurgical Research Institute (CMRDI), Helwan, Egypt. The Data were collected at a scan rate of 1°/min with the scan angle from 5° to 40°. The crystalline index (CrI) was calculated for the three samples using the formula reported by Kaya & Baran, (2015) [28]: CrI = [(I_110_ − I_am_) /I_110_] × 100 (1) Where I_110_ is the maximum intensity at 2θ ≈ 19 and I_am_ is the intensity of amorphous diffraction.

##### Elemental Analysis

A Vario EL III analyzer (Elementar, Hanau, Germany) was used for the analysis of C, H and N contents of the cockroach dried exoskeleton, OSPC, crab and shrimp chitin samples according to the standard operating procedures provided by the manufacturer, at the Micro Analytical Center, Faculty of Science, Cairo University. The degree of acetylation (DA) for the samples was calculated using the formula reported by Kaya & Baran, (2015) [28]: DA= [([C/N] − 5.14) /1.72] × 100 (2)

##### Scanning Electron Microscopy (SEM)

The morphological details of the OSPC, crab and shrimp chitin samples were studied using SEM Model Quanta 250 FEG (Field Emission Gun) attached with EDX Unit (Energy Dispersive X-ray Analyses), with accelerating voltage 30 KV., magnification14 × up to 106 and resolution for Gun.1n. (©FEI company, Eindhoven, The Netherlands), at the Central Laboratories sector of the Egyptian Mineral Resources Authority, Giza, Egypt.

#### 2.1.3. The Chitin Content in the Dried Exoskeleton

The chitin content in the dried exoskeleton was estimated by calculating the mean of the chitin content in 15 samples of the dried exoskeleton. Each sample was refluxed with 50 mL of 4% NaOH at 90 °C for 48 h. The extracted chitin was filtered, washed with distilled water and dried at 150 °C for 2 h. The chitin ratio was calculated according to the following formula: Chitin%= [wt.1/wt.2] × 100 (3) Where wt.1 was the weight of the extracted chitin and wt.2 was the weight of the dried exoskeleton.

### 2.2. Bioethanol Production 

The OSPC (in a colloidal form) was employed as a substratum in a submerged fermentation for bioethanol production. The OSPC was converted to colloidal particles by following the procedure reported previously by Kang, Park, & Lee, (1999) [30]. Fifteen grams of OSPC was dissolved in 100 mL of 10 M HCl at 95 °C in a water bath for 45 s while stirring. The dissolved chitin was then added drop by drop to 2000 mL icy water while stirring and then kept refrigerated at 4 °C for 15 min. The colloidal particles were then collected by vacuum filtration using Whatman filter paper (Grade 5) and washed with distilled water until pH 4 was reached. 

#### 2.2.1. Fungal Strains and Culture Media

##### Fungal Strains and Their Maintenance

Three native fungal strains (Table 1) were used in the present study. They were purchased from the culture collection of the Assiut University Mycological Center (AUMC), Assiut, Egypt. Potato dextrose agar (PDA; Difco, Sparks, MD., USA) was used for the maintenance and sub-culturing of the fungal strains. For inoculum preparation, the three fungal strains listed in (Table 1) were pre-cultivated on PDA slants at 28 °C for 7 days. After cultivation, a spore suspension of each strain was prepared by flooding a fully sporulated culture with a sterilized saline solution (0.9% NaCl) and shaking vigorously for 1 min. Spores were counted by a hemocytometer and the concentration of spores was adjusted to approximately 7 × 10^6^ spore/mL for each strain [31].

##### Production Medium Preparation

The medium used in the submerged fermentation processes of OSPC was prepared according to the method described by Inokuma et al., (2013) [8] with some modifications. The previously prepared suspension of colloidal OSPC (15 g) was suspended in 1liter of distilled water and supplemented with 5 g yeast extract, 1 g peptone, 1 g CaCl_2_.H_2_O, 0.75 g MgSO_4_, 7.5 g NH4Cl, 5 g (NH_4_)_2_SO4 and 4 g KH_2_PO_4_.

#### 2.2.2. Cockroach Chitin Fermentation Mechanisms

Before applying the two fermentation mechanisms, a preceding step of submerged fermentation of OSPC colloidal particles with *T. harzianum* AUMC 5408 was conducted to determine the effect of the initial pH of the production medium on the fungal chitinases production and chitin degradation via estimating the produced NAG titers. *Trichoderma harzianum* AUMC 5408 was subjected to diverse cultural pH values to determine the optimum initial pH value for chitinases production and chitin degradation. The initial pH of the production medium was adjusted to 2, 3, 4, 5, 6, 7 or 8 using 0.1 N HCl or 0.1 N NaOH. A 1 mL of the previously counted spore suspension of *T. harzianum* AUMC 5408 was inoculated into 20 mL of the sterilized production medium in a 100-mL screw cap autoclavable bottle for each pH value (3 replicates were conducted). Some of the media were filtered using Whatman filter papers (Grade 5) and the filtrate was then used for estimation of the produced NAG concentration. The concentration of NAG was estimated according to the per-protocol of Yanai et al., (1992) [32]. The optimum initial pH was then chosen to optimize the proposed fermentation mechanisms for ethanol production.

##### Direct Fermentation Mechanism

One ml of the previously counted spore suspension of each strain of *M. circinelloides* was inoculated in 20 mL sterilized production medium in a 100-mL screw cap autoclavable bottle of a pH 4 (determined in the previous step) and incubated at 28 °C for 6 days with shaking at 120 rpm (3 replicates were conducted for each strain). The media were then filtered and the filtrate was used for bioethanol quantification.

##### Indirect Fermentation Mechanism

*Trichoderma harzianum* AUMC 5408 was used as an aiding chitinolytic agent for biodegradation of OSPC colloidal particles to NAG. A 2.5 mL of the previously counted spore suspension of *T. harzianum* AUMC 5408 was inoculated into 50 mL of the sterilized production medium in a 250-mL screw cap autoclavable bottle of pH value 4 (determined in the previous step) 3 replicates were conducted. All bottles were incubated at 28 °C for 4 days with shaking at 120 rpm. The medium obtained from each bottle was filtered to remove the old mycelia of the grown *T. harzianum* without the removal of the undegraded colloidal chitin particles. The recovered filtrate was re-supplemented with 5 g/L yeast extract, 1 g/L peptone, 1 g/L CaCl_2_.H_2_O, 0.75 g/L MgSO_4_, 7.5 g/L NH_4_Cl, 5 g/L (NH_4_)_2_SO_4_ and 4 g/L KH_2_PO_4_ and its pH value readjusted to pH 4 and re-sterilized. Twenty ml of this medium was inoculated with 1 mL of a spore suspension of each strain of *M. circinelloides* (separately with 3 replicates for each strain) and incubated at 28 °C with shaking at 120 rpm for 48 h. After incubation, the media were filtered and the filtrate was used for bioethanol quantification.

##### Bioethanol Quantification

Bioethanol was quantified at the Micro Analytical Center, Faculty of Science, Cairo University using the YL6500 Gas Chromatograph (YL Instrument Co., Ltd., Anyang, Korea) according to the peer-protocol of Inokuma et al., (2013) [8]. 

##### Statistical Analysis

The obtained data were statistically analyzed using Minitab^®^18 software. One-way ANOVA was conducted to determine the significant differences in the NAG and bioethanol production. Post-Hock analysis (Tukey’s procedure) was conducted to identify sample means that are significantly different from each other (*p* < 0.05).

## 3. Results

### 3.1. Chitin Extraction and Characterization

#### 3.1.1. FT-IR

**Figure 1:**
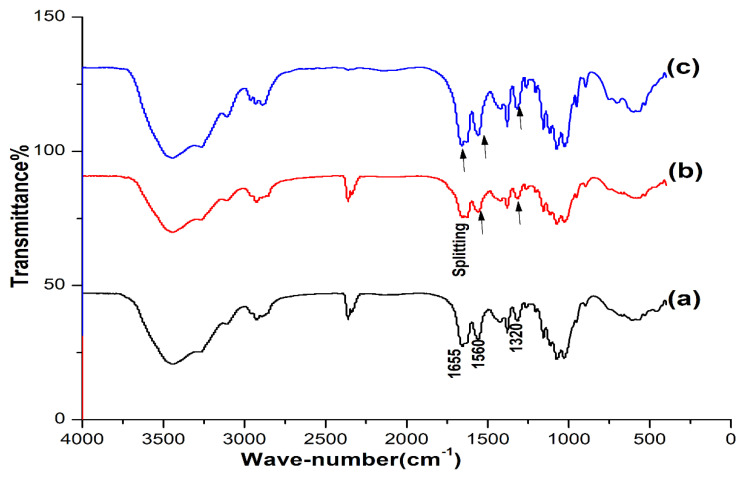
IR spectra of the one-step extracted *P. americana* chitin (**a**), crab chitin (**b**) and shrimp chitin (**c**). High similarity was observed between the three samples.

The IR spectra of OSPC were compared with those of commercial shrimp and crab chitins. Comparing the IR spectra revealed the great similarity between the OSPC, crab and shrimp chitins. The spectra of the three samples were characterized by three amide peaks at wave numbers ≈ 1655, 1560 and 1320 cm^−1^ corresponding to the amide Ι stretching of C=O, the amide ΙΙ of N-H and amide ΙΙΙ of C-N, respectively, with an observed splitting of the amide I into three samples at about 1655 and 1625 cm^−1^ (Figure 1).

#### 3.1.2. XRD

**Figure 2:**
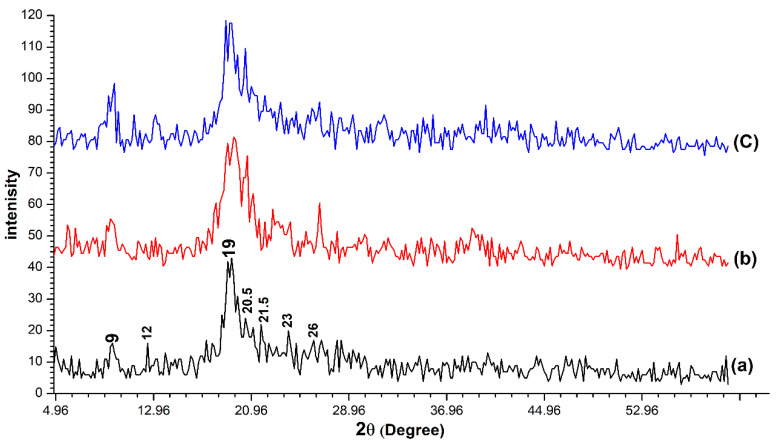
The X-ray diffraction (XRD) patterns for the one-step extracted *Periplaneta americana* chitin (**a**), crab chitin (**b**) and shrimp chitin (**c**). High similarity was observed between the three samples.

The XRD patterns matching for the three chitin samples determined a large similarity between them (Figure 2). The three samples were characterized by two strong peaks at about (2θ ≈ 9, 2θ ≈ 19) and five faint peaks at about (2θ ≈ 12, 2θ ≈ 20.5, 2θ ≈ 21.5, 2θ ≈ 23, 2θ ≈ 26). The crystallinity index (CrI) was calculated according to formula (2) to be 85.7%, 82.5% and 83.72% for OSPC, crab chitin and shrimp chitin respectively.

#### 3.1.3. Elemental Analysis

The C, H and N contents and the calculated DA are clarified in (Table 2). The obtained data revealed a rapprochement in DA values between the OSPC, crab and shrimp chitins. The DA was also observed to drop from 306.3% for *P. americana* dried exoskeleton to 103% of the OSPC.

#### 3.1.4. SEM

**Figure 3:**
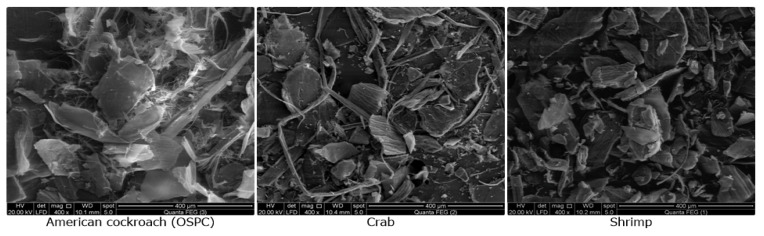
Scanning electron microscopy (SEM) pictures of the three chitin samples, the general theme revealed chitin flakes and microfibers in the three samples with much-isolated fibers observed for the OSPC.

**Figure 4:**
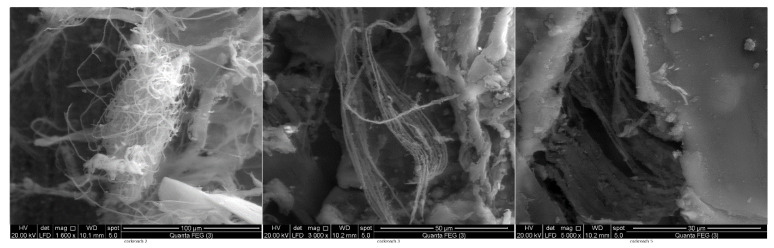
SEM of the one-step extracted *P. americana* chitin (OSPC) flakes at different magnification powers revealing the nanofibers composing the surface of the flake.

Scanning the surface morphology of OSPC and the two commercial samples revealed the large similarity between the three samples. All samples consisted mainly of chitin flakes and micro chitin fibers. The OSPC revealed large numbers of isolated micro chitin fibers if compared to the two commercial samples (Figure 3) also the magnified flake surfaces were found to consists mainly of nanofibers (Figure 4).

##### Chitin Content in the Dried Cockroach Exoskeleton

The chitin content in the dried exoskeleton of *P. americana* was estimated to be about 20%.

### 3.2. Effect of Initial pH on Chitin Degradation

The statistical analysis of the produced NAG concentrations revealed the influence of the initial pH value of the production medium on chitin degradation (Table 3), as the produced NAG was significantly different with a *p*-value 0.0001 (<0.05). *Trichoderma harzianum* AUMC 5408 produced maximum NAG yield (12.785 ± 0.77 g/L) at initial pH 4, followed by pH 3 (10.545 ± 1.3 g/L) with no significant difference between the amount of NAG produced in media adjusted with these two initial pH values. Beyond these pH values, NAG production was significantly decreased. These findings suggest that the fungal chitinases responsible for chitin degradation to NAG are stimulated by acidic pH values.

### 3.3. Bioethanol Production

In the direct fermentation mechanism, after 6 days of shaking incubation, the estimated ethanol titter showed a significant difference between the ethanol productivity of the two tested *Mucor* strains (Table 4). *M. circinelloides* AUMC 6017, produced 11.22 ± 0.562 g/L of ethanol (74% of the initial OSPC loaded in production medium were bio-converted to ethanol). While *M. circinelloides* AUMC 6027 produced 1.94 ± 0.1484 g/L of ethanol (13% of the initial OSPC loaded in production medium were bio-converted to ethanol).

In the indirect mechanism, *M. circinelloides* AUMC 6017 also showed potential for bioethanol production when compared with *M. circinelloides* AUMC 6027. Both the bioethanol productivity of *M. circinelloides* AUMC 6017 and *M. circinelloides* AUMC 6027 from the OSPC colloidal particles was slightly improved (Table 4) when *T. harzianum* AUMC 5408 used as an aiding chitinolytic agent. The bioethanol produced from 15 g/L of the initial OSPC using AUMC 6017 reached 11.92 ± 0.187 g/L after 6 days of incubation (79.5% vs. 74% in case of the direct pathway). AUMC 6027 produced 2.68 ± 0.242 g/L of bioethanol (17.9% vs. 13% in the case of the direct pathway). statistically, there was no significant difference between direct and indirect fermentation mechanisms using the two tested Mucor strains. Generally, *M. circinelloides* AUMC 6017 showed obvious potential over the *M. circinelloides* AUMC 6027 in bioethanol production (Table 4).

## 4. Discussion

Chitin, the main constituents of arthropods’ exoskeleton, have recently drawn the attention of many industrial and medical applications [22,33]. The conventional method for chitin extraction from arthropods’ exoskeleton is a multi-step chemical processing, depends mainly on two steps: demineralization by treating the exoskeleton with hot mineral acids and deproteinization by further treating with hot alkali [22,24,34]. In some cases, additional treatments are used to enhance the purity of the extracted chitin through the removal of fats and pigments using organic solvents and oxidizing agents, respectively [22,24,29,34,35]. However, insect cuticle is less mineralized than that of crustaceans, this facilitates their demineralization [28,36]. Thus, time, cost and chemical wastes related to crustacean exoskeletons processing for chitin extraction can be reduced [24,37]. Based on the nature of insect integument the present study proposed a simple extraction technique using a deproteinization step only regarding the purity of the extracted chitin. Since the purity of the extracted chitin is relative value depends mainly on the extraction procedure [28] the physicochemical properties of OSCP were compared to some purified commercial products of shrimp and crab chitins to assess its purity.

Three crystalline forms of chitin may exhibit according to the different orientations of its microfibrils: α-chitin (anti-parallel chains), β-chitin (parallel chains) and γ-chitin (the combination of parallel and anti-parallel chains) [38]. The IR characteristics of the OSCP were matched those of the commercial products with high similarity indicating the efficiency of the simple extraction technique in isolating chitin from the cockroach exoskeleton. Splitting the amide, the I band indicated that the three samples were in the α-form. The same results for *P. americana* wing-chitin extracted using the conventional method were reported by Kaya & Baran [28]. The XRD patterns of the OSCP were also matched these of the commercial products which means that the simple extraction technique was efficient in isolating chitin from the cockroach exoskeleton without affecting its crystallinity. The same XRD results for *P. americana* chitin extracted using the conventional method were reported by Kaya & Baran, (2015) and Wanule et al., (2014) [28,29]. The calculated CrI according to Formula (2) was 85.7% for the OSPC. It was relatively higher than that of the reference commercial samples. However, the CrI for the OSPC was nearly the same value reported by Kaya & Baran, (2015) [28] as they estimated the CrI of *P. americana* wing-chitin to be 86.7%. The CrI values of chitin from different organisms can vary in the range of 54–90% [24,39] this wide range can be attributed to differences in chitin source and purity of the isolated chitin [28]. Estimating the C, H, N content of OSPC also indicated its relative purity as compared with the usage of pure commercial samples. The DA of OSPC was calculated to be 103% for the whole-body chitin, the rapprochement of this value with values of commercial samples indicated the effectivity of the applied extraction technique in chitin isolation. The estimated DA for OSPC has also matched the DA value of *P. americana* chitin extracted from wings using the conventional method reported by Kaya & Baran, (2015) [28] as they estimated the DA to be 98.67%. However, several previous works reported that the DA of chitin from shrimp, insect and crab were 112%–237%, 101%–132% and 104% respectively [24,36,37]. These values were higher than 100% (the percentage of complete acetylated chitin), indicating that the extracted chitin contains some impurities, such as proteins and inorganic compounds [28]. Dropping the DA from 306.3 % for the dried exoskeleton to 103% for the OSPC also confirmed the efficiency of the conducted extraction technique in the removal of most integument proteins and inorganic compounds.

The estimated chitin content was a rational percentage for an insect exoskeleton since most of the previous works estimated the insect chitin content from 15%–36% [24,36,39]. However, it was relatively higher than that reported by Kaya & Baran, (2015) [28] who estimated 18% chitin in wings and 13% for other body parts of *P. americana* while, Wanule et al., (2014) [24] reported 24% chitin in *P. americana* exoskeleton. These differences in measured chitin content for the same organism may be related to the method of extraction [28]. Generally, the results of OSPC characterization revealed that the deproteinization of the dried exoskeletons alone is sufficient for chitin extraction and purification. This is due to the nature of insect integument since it is characterized by low levels of inorganic materials (less than 10%) as compared to crustacean shells (20%–40%) [24]. Although, using crab and shrimp shell wastes yields more chitin (20%–40%) [22] if compared to *P. americana.* The simple chitin extraction technique, the ease of mass-rearing and the short life cycle may be some potential factors that act in favor of *P. americana* as a chitin source. *Thus, P. americana* could be a promising alternative source for production of low cost and highly pure chitin.

Scanning the surface morphology also revealed a matched general-characteristics of OSPC with the commercial samples since they all consist mainly of chitin flakes and microfibers. According to previous studies, chitin flakes may attain three surface morphologies, which are (1) hard and rough flakes without pores or nanofibers, (2) flakes solely composed of nanofibers and (3) flakes with both pores and nanofiber [28]. According to the present study, OSPC was characterized mainly by flakes surface composed of nanofibers. Kaya & Baran, (2015) [28] described chitin flakes only has oval nanopores (230–510 nm) without nanofiber from *P. americana* wings. However, the chitin fibers were thought to be easily degraded enzymatically than chitin crystals [40] thus OSPC may show easily enzymatic degradation if used in the bioconversion process.

Chitin, like lignocellulosic biomass, needs a multi-step pathway for bioconversion to ethanol. This multi-step process starts with chitin pretreatment, microorganism chitinases production, enzymatic degradation of chitin to NAG and fermentation of NAG [8,21]. The bioconversion process depends mainly on the chitinolytic activity of the used microorganisms. Chitinolytic enzymes of microorganisms may contain endochitinases, exochitinases, chitobiosidases and N-acetyl-d-glucosaminidases (NAGases). The chitinolytic activity of microorganisms starts by increasing the solubility of chitin as the first step in its biodegradation process. The endochitinases within microorganisms cleave chitin randomly generating the soluble and low molecular weight oligomers. Then the exochitinases (chitobiosidases in some organisms), catalyze the cleavage of dimers. Finally, the produced oligomers and dimers by endochitinases and exochitinases are hydrolyzed by NAGases, to generate NAG [41] which in turn ferments easily to bioethanol. The commercial application of any biomass in the bioconversion process for fuel production always seeks a reduction in steps of fermentation to reduce both cost and waste. Thus, the presence of microorganisms can bio-convert chitin directly to ethanol may be a potential solution to facilitate using the untapped chitinous biomass as a bioethanol source, in turn replacing the energy crops in fuel production.

Inokuma et al., 2012 [8], in their study, introduced some strains of fungus *Mucor circinelloides* to produce bioethanol using shrimp chitin as substrates in one step. These strains produced chitinolytic enzymes and could convert chitin substrate into bioethanol directly under microaerobic conditions in submerged fermentation systems. However, the observed ethanol production was low due to a shortage of *M. circinelloides* chitinolytic enzyme activity. Therefore, to commercialize ethanol production using *M. circinelloides* strains, they suggested optimizing all aspects of fermentation, screening of chitinases for addition, construction of genetically engineered Mucor strain and the establishment of effective chitin pretreatment method. Based on these results and suggestions the present study aimed to test the possibility of using the OSPC as a substrate in submerged fermentation systems with native strains of *Mucor circinelloides* for bioethanol production. Also, to test some aspects which may affect the bioconversion process. First, the OSPC pretreatment to colloidal chitin was suggested since, colloidal chitin was found to be easily degraded enzymatically [8,40] second, instead of constructing genetically engineered microorganism an *M. circinelloides* strain isolated from a chitinous source was suggested to have the potential ability in chitin degradation and fermentation directly to ethanol. Third, instead of using chitinases for addition to the bioconversion pathway, a common chitinolytic fungus was suggested to be used as an aiding chitinolytic agent to enhance the ethanol production via degrading chitin to NAG and following the produced NAG in further fermentation with *M. circinelloides*. Fourth, since, chitin degradation can be conducted chemically, enzymatically (via the action of purified chitinolytic enzymes) or by using biotransformation methods using the entire chitinolytic microorganism for biodegradation [40] Also, an efficient chitin degradation and NAG production by biotransformation using the whole microorganism was reported earlier [40,42,43,44]. All fermentation processes were suggested to be conducted using the whole microorganism growing in the production medium to simplify the fermentation and decrease the bioconversion steps. Finally, the pH parameter was chosen to optimize the process of chitin degradation because production and the activity of the fungal chitinases and chitin degradation, were reported to be dramatically influenced by the pH value [45] regarding constant fermentation conditions reported previously in the study by Inokuma et al., 2012 [8].

These aspects were studied by constructing two fermentation mechanisms. First, a one-step fermentation process (a direct mechanism), was achieved by testing two native strains of *M. circinelloides* in a direct submerged fermentation process. The two strains were chosen based on their source of isolation one of them isolated from a chitinous source, the honey bee and the other was isolated from a non-chitinous source, the cow dung. The second mechanism, a two-step bioconversion process, starts by submerged chitin degradation with familiar chitinolytic fungal strain, to enhance chitin degradation to NAG which was found to be more readily available for bioethanol production than the colloidal chitin [8]. The produced NAG was then followed in the second step of fermentation with the same two *M. circinelloides* used in the direct fermentation process. *Trichoderma harzianum* AUMC 5408 was chosen because of its potential chitinolytic activity [46,47,48].

The results of the two constructed fermentation processes supported most of our proposed approaches as follows; first, detection of both ethanol and NAG in the media filtrate confirmed the possibility of using the whole microorganisms in bioconversion of chitin means a simplified bioconversion processing is possible. These results concurred with some previous studies [8,40,42,43,44]. Second, the chitin degradation and production of fungal chitinases were shown to be influenced by the initial pH of the production medium. Chitin degradation using fungal chitinases was optimized under acidic conditions these results also, are in accordance with various previous works [45,47,48,49,50,51,52,53]. Third, *M. circinelloides* AUMC 6017 of chitinous isolating source showed a high ethanol production rate than the non-chitinous source-isolated strain, *M. circinelloides* AUMC 6027 whatever the used fermentation mechanism. According to the direct fermentation mechanism, *M. circinelloides* AUMC 6017 could biotransform about 74% of the initial substrate (OSPC colloidal particles) within 6 days of shaking incubation. It significantly differed from that produced by *M. circinelloides* AUMC 6027 which could biotransform about 13% of the initial substrate (OSPC colloidal particles) in 6 days under the same conditions of incubation. Inokuma et al. (2013) [8], reported NBRC4572 (NITE Biological Resource Center, Japan) among many strains of *M. circinelloides* all are of non-chitinous isolating sources according to the NBRC to produce highest ethanol titers directly using shrimp chitin colloidal particles as substrate. This non-chitinous source isolated strain (Kaoliangchiu yeast cake) could bio convert about 12% of the initial substrate (50 g/L) in 16 Days of the same incubating conditions. Regarding the time of incubation and the amount of ethanol produced, it is obvious that *M. circinelloides* AUMC 6017 shows potential activity in direct ethanol production using colloidal chitin as a substrate over these non-chitinous strains. These results reinforced the suggestion of a fungal isolation source to be responsible for this potent activity, since *M. circinelloides* AUMC 6017 may attain an evolutionary adaptation in chitin degradation and fermentation owing to its evolutionary relationship with the insect chitin. The indirect mechanism also revealed the same conclusion. In total *M. circinelloides* AUMC, 6017 could biotransform about 79.5% of the initial substrate (OSPC colloidal particles) within 6 days of shaking incubation. It significantly differed from that produced by *M. circinelloides* AUMC 6027 which could biotransform about 17.9% of the initial substrate (OSPC colloidal particles) in 6 days. Inokuma et al. (2013) [8] reported that by using the concentrated chitinolytic enzymes of *Mucor ambiguus* NBRC 8092, the chitin bio-transformed to ethanol by their native strain of *M. circinelloides* NBRC 4572 improved from only 12% to 18.9 % after 16 days of incubation. These findings highlighted *M. circinelloides* AUMC 6017 (honeybee isolating source) as a novel potential strain for direct bioethanol production from the insect colloidal chitin as a substrate. Fourth, the pH of the production medium was also showed to affect the time of bioconversion besides the source of isolation of the fungal strains.

## 5. Conclusions

*Periplaneta americana* is a cosmopolitan insect species that can be cultured easily on different organic food sources and can yield large biomass in small areas of production. As the insect cuticle has low inorganic materials, the deproteinization processing of the dried cockroach exoskeleton is sufficient for the extraction and purification of cuticular chitin. Thus, insects could be considered as an alternative low-cost chitin source, that extracted chitin can be easily bio-convert directly to ethanol in a simple submerged fermentation system using a native strain of *M. circinelloides* (AUMC 6017) isolated from a chitinous source, the honey bee. In our next studies, we aim to test the genetic variability and compare more fungal strains of the chitinous isolating source with strains of the non-chitinous isolating source to confirm our conclusions. Optimizing all the fermentation conditions and testing chitins of diverse sources are also, important to commercialize the bioconversion of chitin biomass for ethanol production.

## Figures and Tables

**Table 1:** Fungal strains used in the present study.

Fungal Strain	AUMC Number	Source of Isolation
*Trichoderma harzianum* Rifai	AUMC 5408	Soil of *Lens esculentus* (lentil) plantation Assiut, 2009
*Mucor circinelloides* van Tieghem	AUMC 6017	Honeybee, Egypt, 2009
*Mucor circinelloides* van Tieghem	AUMC 6027	Cow dung, Sohag, 2009

**Table 2:** The C, H, N analysis and the calculated degree of acetylation (DA) of *P. americana* dried exoskeleton (PDEX), the one-step extracted *P. americana* chitin (OSPC), crab chitin and shrimp chitin.

Sample	C%	H%	N%	DA%
* PDEX	32.48	5.74	3.12	306.3
* OSPC	43.84	6.93	6.33	103
Crab chitin	43.49	6.99	6.12	107.6
Shrimp chitin	43.32	6.85	6.41	94

* PDEX: *P. americana* dried exoskeleton, * OSPC: one-step extracted *P. americana* chitin.

**Table 3:** The mean N-acetyl-d-glucosamine (NAG) produced by *T. harzianum* at different initial pH values.

pH	* N	Mean NAG * (±STD) g/L	Grouping			
4	3	12.785 ± 0.77	A			
3	3	10.545 ± 1.3	A			
6	3	7.549 ± 0.85		B		
5	3	5.950 ± 0.23		B	C	
7	3	4.335 ± 1.13			C	D
2	3	2.1425 ± 0.56				D
8	3	1.965 ± 0.23				D

* Means that do not share a letter are significantly different, * STD: Standard deviation, * N: number of replicates.

**Table 4:** Bioethanol production by two *Mucor circinelloides* strains using the one-step extracted *Periplaneta americana* chitin as a substrate.

Strain/Fermentation Mechanism	* N	Mean Bioethanol * (±Std) g/L	Grouping	
*M. circinelloides* AUMC 6017/Indirect	3	11.92 ± 0.06807	A	
*M. circinelloides* AUMC 6017/Direct	3	11.22 ± 0.312	A	
*M. circinelloides* AUMC 6027/Indirect	3	2.68 ± 0.03512		B
*M. circinelloides* AUMC 6027/Direct	3	1.94 ± 0.05033		B

Note: Means that do not share a letter are significantly different, * Std: Standard deviation, * N: number of replicates.
